# Impact of immune checkpoint inhibitors on vision and eye health

**DOI:** 10.1038/s41433-024-03212-z

**Published:** 2024-07-03

**Authors:** Mouayad Masalkhi, Noura Wahoud, Bridget Moran, Ezzat Elhassadi

**Affiliations:** 1 School of Medicine, University College Dublin, Belfield, Dublin Ireland; 2 Faculty of Medicine, American University of Beirut, Beirut, Lebanon; 3 Mater Miscordiae University Hospital, Dublin, Ireland; 4 Haematology Department, University Hospital Waterford, Waterford, Ireland

**Keywords:** Immunosuppression, Immunopathogenesis, Chemotherapy

## Introduction

Immune checkpoint inhibitors (ICIs) have emerged in the last decade as a groundbreaking class of drug in the management of different cancers [1]. ICIs activate the immune system to attack tumour cells by blocking their 2 immune inhibitory signals such as Cytotoxic T-lymphocyte associated protein 4 (CTLA-4), programmed death-1 (PD-1) or its partner programmed death-1 ligand (PDL-1) receptors [2]. Although this mechanism has been of therapeutic benefit, unopposed immune activation leads to off-target ‘immune-related adverse events” (IRAEs) that could affect multiple organ systems [1, 3, 4]. Although ocular IRAEs are rare, they can be sight-threatening [5]. In this paper, we discuss the impact of immune checkpoint inhibitors on vision along with their diagnosis and management.

## Ocular IRAEs of immune checkpoint inhibitors

Ophthalmic toxicities related to ICI use are relatively uncommon, occurring in approximately 1% of patients, with dry eye disease (DED), conjunctivitis, and keratitis being the most frequently documented manifestations [6, 7]. Most of these cases were mild to moderate in severity, resolving spontaneously or with conservative treatment [6, 7]. Other less commonly reported adverse events include corneal perforation, corneal ulcer, pseudomembrane formation, blepharitis, episcleritis, persistent epithelial defect, and corneal graft rejection [2]. The infrequency of these adverse events may be attributed to the immunologically privileged protective environment of the eye with the most prominent theories suggesting that regulatory T cells (Treg) cells may suppress autoreactivity [8].

The time interval between the use of ICI and onset of DED symptoms widely varies from days to months, with symptoms including foreign body sensation, red eye, irritation, blurry vision, and photophobia [8]. Dry eye was reported with the use of Ipilimumab, Nivolumab, Pembrolizumab, Atezolizumab, Avelumab, and Durvalumab [2, 9, 10]. The pathophysiology of ICI-induced DED could be attributed to lacrimal gland dysfunction secondary to the autoimmunity induced by these agents. Another theory hypothesises that ICI causes granulomatous inflammation of the lacrimal glands like that seen in sarcoidosis through CD8 + T cells infiltration and secretion of IL-2 [11]. In addition to ocular surface pathologies, ICI use has been associated with a variety of neuro-ophthalmic complications involving both the central and peripheral nervous system, including optic neuropathy, orbital inflammatory disease, and ocular myasthenia [12]. While corticosteroids are typically used to manage immune-related adverse events, there is no consensus on whether they should be used in the management for neuro-ophthalmic adverse events or whether ICI immunotherapy should be stopped altogether [12].

In a cohort of 41,674 patients, ocular AEs comprised 3.0% of all ICI reports with uveitis being the most prevalent (15.1%) followed by retinal (10.7%), lacrimal (9.0%), optic nerve (8.4%), and conjunctival (8.2%) disorders [13]. The most common indications for ICI therapy were lung cancer and melanoma, comprising 27.3% and 22.7% of cases respectively [13]. Uveitis and retinal disorders induced by ICI were more strongly associated with melanoma than lung cancer [13]. This could be explained by antigen sharing between uveal melanocytes and melanoma cells, such as gp100 and tyrosinase [13]. It has been extensively demonstrated in the literature that anti-CTLA-4 therapy is more toxic than anti-PD-1 or anti-PD-L1 treatment, with the most severe systemic toxicity occurring with their combination [13]. The combination of anti-CTLA4 and anti-PDL-1 therapy resulted in a 1.5-3.5-fold increase in each ocular AE compared to anti-PD-1 therapy alone. Of note, ipilimumab (anti-CTLA-4), was reported to have an irAE rate of 60–65% of any grade with over 40% of patients developing serious irAEs (grade 3–4) compared with lower grade and less frequent irAEs in the anti-PD-1 and anti-PD-L1 monotherapy groups [13]. Moreover, the combination of ipilimumab with nivolumab, an anti-PD-1 agent, resulted in 59% rate of grade 3-4 irAEs compared to a rate of 21–28% rate in the anti PD-1 monotherapy group [13].

In a separate paper by Noble et al., ocular IRAEs ranged from intraocular inflammation (anterior, intermediate, posterior, or panuveitis) to keratoconjunctivitis sicca (dry eye syndrome) and optic neuropathy with symptoms developing at a mean of 15.7 weeks from onset of therapy [14]. While symptoms were generally well-controlled with corticosteroids, 6 out of 11 patients had to discontinue treatment due to decreased visual acuity, progression of cancer metastasis or severe systemic irAEs [14].

Blepharitis and dry eye disease were reported in a single case 9 weeks after starting pembrolizumab therapy [14]. Symptoms were successfully managed with artificial tears and conservative measures [14]. Intermediate uveitis was also reported with pembrolizumab, and responded well to topical corticosteroids [14]. Nongranulomatous anterior uveitis (NGAU) developed in 75% of patients receiving nivolumab [14]. Although symptoms were managed with topical corticosteroids, 1 patient needed IV corticosteroids for a sixth cranial nerve palsy and 2 patients had to discontinue treatment due to decreased visual acuity [14]. Choroidal inflammation as part of a Vogt-Koyanagi-Harada (VKH)-like reaction was reported in 3 melanoma patients receiving either Pembrolizumab, Ipilimumab, or Ipilimumab and Nivolumab combination therapy [14]. All three patients had resolution of choroidal inflammation with oral and topical corticosteroids [14]. Optic neuropathy was reported in a case of durvalumab-treated prostatic cancer with significant improvement in symptoms with intravenous high dose corticosteroids [14]. Table 1 outlines the ocular adverse effects associated with various immune checkpoint inhibitors as reported by different studies, along with the types of ocular adverse effects noted (like dry eyes, blepharitis, uveitis, optic neuropathy, and ocular myasthenia).

**Table 1:** Ocular adverse effects reported with various immune checkpoint inhibitors (with their mechanisms of action).

Authors	Mechanism of action/target receptor	The specific immune checkpoint inhibitor(s)	Ocular Adverse effect	Reference
Noble et al.	Programmed Death-1 receptor inhibitor	Pembrolizumab	Dry Eyes, Blepharitis, Intermediate Uveitis	[14]
	Programmed Death-Ligand 1 receptor inhibitor	Durvalumab	Non-Granulomatous Anterior Uveitis, Optic neuropathy	
	Programmed Death-1 inhibitor	Nivolumab	Non-Granulomatous Anterior Uveitis	
	Cytotoxic T-Lymphocyte-Associated Protein 4 and Programmed Death-1 inhibitor	Ipilimumab and Nivolumab	Non-Granulomatous Anterior Uveitis, Vogt-Koyanagi-Harada-like reaction	
	Cytotoxic T-Lymphocyte-Associated Protein 4 receptor inhibitor	Ipilimumab	Vogt-Koyanagi-Harada-like reaction	
Zhang et al.	Cytotoxic T-Lymphocyte-Associated Protein 4 inhibitor	Ipilimumab	Uveitis	[5]
	Cytotoxic T-Lymphocyte-Associated Protein 4 inhibitor	Tremelimumab		
	Programmed Death-1 inhibitor	Nivolumab		
	Programmed Death-1 receptor inhibitor	Pembrolizumab		
	Programmed Death-1 inhibitor	Atezolizumab		
Bomze et al.	Programmed Death-1 inhibitor	Ipilimumab	Uveitis disorders Vision disorders Retinal disorders	[13]
	Programmed Death-1 inhibitor	Nivolumab		
	Programmed Death-1 receptor inhibitor	Pembrolizumab		
	Programmed Death-1 inhibitor	Atezolizumab		
	Programmed Death-1 inhibitor	Durvalumab		
	Programmed Death-Ligand 1 inhibitor	Avelumab		
Fang et al.	Programmed Death-1 inhibitor	Atezolizumab	Uveitis Eye inflammation	[16]
	Cytotoxic T-Lymphocyte-Associated Protein 4 inhibitor	Ipilimumab	Uveitis Dry eye Eye inflammation	
	Programmed Death-1 inhibitor	Nivolumab	Uveitis Dry eye Ocular myasthenia Eye inflammation	
	Programmed Death-1 inhibitor	Pembrolizumab	Uveitis Ery eye Ocular myasthenia	

## Diagnosis and management of ocular irAEs

Uveitis represents a significant majority of ocular immune-related adverse events (IRAEs), making up about 70% of these conditions when associated with the use of ICIs [5]. Diagnostic efforts to confirm ICI-induced uveitis include tools such as the Naranjo criteria, which help to differentiate it from other causes [5]. Accurate diagnosis also requires comprehensive laboratory investigations to rule out other potential infectious or autoimmune causes [5]. This involves tests such as serologic assessments for antinuclear antibodies (ANA), antineutrophil cytoplasmic antibodies (ANCA), dsDNA antibodies, and rheumatoid factor [5]. Serum angiotensin-converting enzyme (ACE) is used as a marker to rule out sarcoidosis associated uveitis which manifests in 30–70% of cases [5]. Infectious causes of uveitis include ocular Epstein–Barr virus (EBV), toxoplasmosis, herpes simplex, syphilis, Lyme disease, and tuberculosis. Serological testing such as fluorescent treponemal antibody absorption test (FTA-ABS), Treponema pallidum particle agglutination (TP-PA), rapid plasma regain (RPR) can be used to diagnose syphilis whereas QuantiFERON-TB is considered the gold standard for diagnosis of TB [5]. Electroretinography (ERG) is a valuable tool that can be used to differentiate ICI-induced ocular IRAEs from melanoma-associated retinopathy [5]. American Society of Clinical Oncology guidelines for ICI-induced uveitis, iritis or episclerites recommendations include continuation of ICI and artificial tears for grade 1, topical corticosteroids with temporal holding of ICIs for grade 2, systemic corticosteroids and permanent discontinuation of ICI for grades 3 and 4 with the consideration of infliximab or other TNF-a blockers in refractory cases [15].

## Conclusion

In conclusion, ocular IRAEs are rare with the majority resolving with conservative management. However, it is important to keep in mind that they can potentially be sight threatening and require the discontinuation of ICI. Regular ophthalmologic follow ups are necessary to ensure ICI safety and future research should focus on understanding the mechanisms driving ocular irAEs and developing more effective management strategies.
